# Involvement of Band3 in the efflux of sphingosine 1-phosphate from erythrocytes

**DOI:** 10.1371/journal.pone.0177543

**Published:** 2017-05-11

**Authors:** Makoto Kurano, Masako Nishikawa, Hiroyuki Kuma, Masahiro Jona, Yutaka Yatomi

**Affiliations:** 1 Department of Clinical Laboratory Medicine, Graduate School of Medicine, The University of Tokyo, Tokyo, Japan; 2 Department of Clinical Chemistry, Faculty of Pharmaceutical Sciences, Nagasaki International University, Nagasaki, Japan; 3 Department of Clinical Laboratory, The University of Tokyo Hospital, Tokyo, Japan; Universidade do Minho, PORTUGAL

## Abstract

Sphingosine 1-phosphate (S1P) is a bioactive lipid mediator that is thought to be involved in various diseases. Although the main source of S1P in the plasma is erythrocytes, how S1P is exported from erythrocytes has not been elucidated. When we differentiated K562 cells into erythroblast-like cells with sodium butyrate, we observed that the efflux of S1P was increased without increased expression of previously proposed S1P transporters, while the expression levels of Band3 were increased. Therefore, in this study, we investigated the involvement of Band 3, the most characteristic membranous transporter for erythrocytes, in S1P efflux, using 4,4'-diisothiocyanatodihydrostilbene-2,2'-disulfonic acid, disodium salt (H2DIDS), which is an inhibitor of Band3. First, we treated human washed erythrocytes with H_2_DIDS and found that H_2_DIDS decreased the S1P levels in the supernatant, while it increased the cellular S1P contents. Next, when we injected H_2_DIDS into mice, the plasma S1P level was significantly decreased. Finally, when we overexpressed or suppressed Band3 in K562 cells, S1P efflux was enhanced or decreased, respectively, while the overexpression of Band3 in HEK293 cells did not modulate S1P efflux. These results suggested the possible involvement of Band3 in the transport of S1P, a multi-functional bioactive phospholipid, from erythrocytes.

## Introduction

Sphingosine 1-phosphate (S1P) is a potent lipid mediator that has various biological properties [1], such as anti-apoptosis [2], cell proliferation [3], vasorelaxation [4], and lymphocyte attraction [5]. The main sources of S1P in the circulation are considered to be erythrocytes [6], platelets [7], and the endothelium [8,9]. Considering the net volumes of erythrocytes and platelets in the circulation, erythrocytes might mainly contribute to circulating S1P, at least in a steady state. In addition, a recent report has demonstrated the biological importance of S1P released from erythrocytes: erythrocytes-derived S1P is essential for vascular development [10]. Therefore, elucidating how S1P is produced and secreted from erythrocytes is an important task.

The intracellular production of S1P is well elucidated (S1 Fig); S1P is produced through sphingosine kinase 1 or sphingosine kinase 2 from its precursor sphingosine and is degraded into sphingosine through S1P phosphatase or into 2-hexadecenal and phosphoethanolamine through S1P lyase (Sgpl) [11]. Since Sgpl is absent in erythrocytes [12], S1P is abundantly stored in erythrocytes. Concerning the mechanism by which S1P is secreted from cells, the secretion of S1P from platelets has been speculated to occur via a vesicle-mediated or exocytotic manner [13] or via some S1P transporters [14], while spinster homolog 2 has been shown to function as an S1P transporter in endothelium cells [9]. However, the mechanism by which S1P is released from erythrocytes has yet to be fully elucidated, although ATP binding cassette subfamily A member 1 has been proposed as a possible candidate [15].

Considering the importance of S1P in the pathogenesis of various diseases [16], in this study, we aimed to elucidate the mechanism involved in the secretion of S1P from erythrocytes and investigated the modulation of S1P homeostasis by examining the differentiation of K562 cells into erythroblast-like cells [17].

## Materials and methods

### Cells

Erythrocytes were obtained from healthy volunteers after obtaining written informed consent; the samples were collected using EDTA-containing glass vacutainer tubes and washed with PBS three times to prepare washed erythrocytes. The study was approved by the Institutional Research Ethics Committee of the Faculty of Medicine, The University of Tokyo.

K562 cells were obtained from the Japanese Collection of Research Bioresources Cell Bank (JCRB, Osaka, Japan) and HEK293 cells were from ATCC (Manassas, VA). K562 cells and HEK293 cells were cultured in RPMI 1640 (R8758, Sigma-Aldrich Co.) and DMEM (D5796, Sigma-Aldrich Co.), respectively, supplemented with 10% fetal bovine serum (FBS, 10099–141; Gibco BRL, Eggstein, Germany) and 1% penicillin/streptomycin (15070–063; Gibco BRL).

### Differentiation of K562 cells

To differentiate the K562 cells into erythroblast-like cells, we treated K562 cells (at a concentration of 5 x 10^6^ cell/mL) with 2 mM sodium butyrate (NAB, 193–01522; WAKO Pure Chemical Industries, Osaka, Japan) [18,19]; 72 hours later, we performed the experiments.

### Effects of H_2_DIDS on S1P and C_17_S1P excretion from cells

After being washed with PBS, the erythrocytes (1.3 x 10^9^ / 4 mL) or K562 cells (2–4 x 10^6^ / 1 mL) were treated with H_2_DIDS (D-338; Invitrogen Co.) dissolved in PBS at a concentration of 0.1 mM at 37°C for 1 hour. Then, the erythrocytes or K562 cells were washed three times with PBS containing 0.5% fatty acid-free BSA. To examine the C_17_S1P excretion, we further treated the cells with 10 μM C_17_-sphingosine at 37°C for 20 minutes. Then, we replaced the supernatant with PBS containing the indicated acceptor and incubated the cells at 37°C for another 20 minutes. The supernatants and cells were then collected and used to measure the C_17_S1P and S1P levels. The acceptors utilized in the experiments were prepared as follows: fatty acid-free albumin was obtained from Sigma-Aldrich Co. and dissolved in PBS at 0.5%, HDL was prepared using a standard ultracentrifugation method and dissolved in PBS at 10%, plasma (obtained from healthy subjects) was dissolved in PBS at 10%, and apoM-rich lipoprotein or control lipoprotein (prepared by condensing the conditional medium of HepG2 cells infected with apoM-coding adenovirus or control adenovirus using Amicon Ultara-15 [UFC901008, Millipore Co., Bedford, MA], as described in a previous paper [20]) was dissolved at 5% in PBS.

### Effects of overexpression or knockdown of proteins on C_17_S1P excretion in K562 cells and HEK293 cells

To overexpress Band3, we cloned human Band3 cDNA obtained from Flexi ORF Clone (Promega, Co., Madison, WI) into a CMV shuttle vector (Stratagene, La Jolla, CA). Then, we transfected human Band3-coding plasmids or GFP-coding plasmids into K562 cells or HEK293 cells using Lipofectamine LTX with Plus Reagent (15338100; Invitrogen Co., Carlsbad, CA), according to the manufacturer’s protocol. To knockdown Band3, we transfected siRNA against human Band3 or control siRNA at 10 nM (sc-42735, sc-37007; Santa Cruz Biotechnology) into K562 cells utilizing Lipofectamine RNAiMAX (12778–075; Invitrogen Co.); 48 hours later, we treated the cells (K562 cells: 2–4 x 10^6^ / 1 mL, HEK293 cells: around 80% confluency) with 10 μM C_17_-sphingosine (860654P; Avanti Polar Lipids, Alabaster, AL) at 37°C for 20 minutes. Then, we replaced the supernatant with PBS containing 0.5% fatty acid-free BSA (A8806; Sigma-Aldrich Co.) and incubated the cells at 37°C for another 20 minutes. The supernatants and cells were then collected and subjected to the C_17_S1P assay.

### Measurement of S1P and C_17_S1P

The contents of S1P and C_17_S1P in the plasma, medium, and liver were determined using two-step lipid extraction followed by HPLC separation, as described previously [21]. Briefly, samples were sonicated in 3 mL of methanol/chloroform (2:1) with an internal standard for 30 minutes. After adding 2 mL of chloroform, 2.1 mL of 1 mM KCl, and 100 μL of 3 N NaOH, the samples were centrifuged and the alkaline upper phase (3.8 mL) was collected into new tubes, to which 4 mL of chloroform and 200 μL of concentrated HCl were then added. The resulting lower chloroform phases (3.5 mL) formed under these new acidic conditions were collected and evaporated under nitrogen gas and resolved in methanol, followed by HPLC separation using a TSKgel ODS-80TM column (0017202; Tosoh, Tokyo, Japan). For the measurement of the S1P content, we used C_17_-S1P (860641P; Avanti Polar Lipids) as an internal standard, while for the C_17_-S1P content, we used FTY720-phosphate (10006408; Cayman Chemical, Ann Arbor, MI).

### Sphingosine kinase activity assay

We measured the sphingosine kinase (SK) activity as described previously [22]. Briefly, we homogenized cells or liver tissues in 20 mM Tris-HCl (pH7.4), 20% glycerol, 1 mM β-mercaptoethanol, 15 mM NaF, 1 mM EDTA, 1 mM Na_3_VO_4_, 1 mM PMSF, and a protease inhibitor cocktail (Roche). The reaction was initiated by adding 25 μL of 200 μM C17-sphingosine and 25 μL of 20 mM ATP to 300 μg of protein in a final volume of 500 μL. After incubation at 37°C for 20 minutes, the reaction was terminated by adding 50 μL of 1 M HCl and 3 mL of methanol and chloroform (v/v = 2:1), followed by the C_17_S1P measurement.

### Measurement of bilirubin and albumin

Plasma bilirubin levels and albumin levels were measured with Bilirubin Assay Kit, QuantiChrom (DIBR-180, BioAssay System, Hayward, CA) and mouse albumin ELISA Kit (E90-134, Bethyl Laboratories, Inc, Montgomery, TX).

### Reverse transcription and real-time PCR

Total RNAs extracted from cells using the GenElute Mammalian Total RNA Miniprep kit (RTN-70; Sigma-Aldrich) were subjected to reverse transcription using ReverTra Ace qPCR RT Master Mix (FSQ-201; TOYOBO Co., Ltd, Osaka, Japan). Non-quantitative PCR was performed using the primers listed in Table 1. Quantitative PCR was performed using an ABI 7300 Real-Time PCR System (Applied Biosystems) for human SK1 (Hs01116530_g1), human SK2 (Hs01016543_g1), human Sgpl (Hs00187407_m1), human Spp1 (Hs00229266_m1), human Band3 (Hs00978603_m1), human glycophorin A (GYPA) (Hs00266777_m1), human 5-aminolevulinic acid synthase (ALAS) (Hs00163601_m1), human ATP binding cassette subfamily C member 1 (ABCC1) (Hs01561502_m1), human ATP binding cassette subfamily G member 2 (ABCG2) (Hs01053790_m1), human ATP binding cassette subfamily B member 1 (ABCB1) (Hs00184500_m1), and human GAPDH (Hs01060665_g1). The expression levels of the genes of interest were adjusted to those of the endogenous GAPDH mRNA as a control.

**Table 1:** PCR primers utilized in the non-quantitative PCR analysis.

gene	forward primer	reverse primer
**ABCC1**	5’ ctggagaggaggaagggagttca 3’	5’ gcagcacggtgtagaagtagcc 3’
**ABCG2**	5’ gtggaggcaaatcttcgttatt 3’	5’ ttctatgagtggcttatcctgct 3’
**ABCA1**	5’ ggaagttctgggctggtattgtg 3’	5’ aaagtactcacagccaaaccca 3’
**ABCB1**	5’ cctggcagctggaagacaaat 3’	5’ gatgcctgtccaacactaaaagc 3’
**Band3**	5’ tagagctgcgtagagtcttcacc 3’	5’ tttaagtctaggcccttgtagaag 3’
**GAPDH**	5’ tcccatcaccatcttccagg 3’	5’ gaggagtgggtgtcgctgtt 3’
**GYPA**	5’ acttcaggaaccagctcatg 3’	5’ ggagtgtccagtccacatgt 3’
**SPNS2**	5’ ggcatgatcacaggaacactcat 3’	5’ catcgccagctggttcacct 3’

### Western blotting

For the preparation of membranous protein, K562 cells or HEK293 cells were homogenized in 5 mM Tris-HCl (pH 7.5) solution containing 1 mM PMSF and protease inhibitor cocktail (Roche, Mannheim, Germany) and centrifuged at 700 g for 15 min three times. The supernatants were centrifuged at 444,000 g for 60 min, and the pellets were suspended in 50 mM Tris-HCl (pH7.5) solution containing 1% TritonX-100, 5 mM EDTA, 10 mM EGTA, and 1 mM PMSF [23]. Western blotting was performed using 30 μg of the cellular proteins or a volume corresponding to 0.02 μL of plasma according to a standard method. The following antibodies were used: anti-Band3 antibody (ab108414, Abcam), anti-pan cadherin antibody (RB-9036-PU, Neomarkers Inc, Fremont CA), anti-mouse apoM antibody (A00954, GenScript Co, Piskataway, NJ), anti-apoA-I antibody (AB740, Chemicon International Inc., Temecula, CA), and anti-mouse albumin antibody (sc-46293, Santa Cruz Biotechnology).

### Flow cytometry analysis

Flow-cytometric analyses of CD235a (GYPA) expression on the NaB-treated K562 cells were performed with a Beckman Coulter Cytomics FC500 flow cytometer, using CD235a-FITC (catalog# IM2212U; lot# 46; Beckman Coulter Inc, Brea, CA).

### Animal experiments

C57BL/6 mice were purchased from CLEA Japan (Tokyo, Japan). Ten-week-old male mice were divided into three groups and injected with PBS, 0.1 mg of H_2_DIDS, or 0.5 mg of H_2_DIDS. The mice were anesthetized by an intraperitoneal injection of sodium pentobarbital (Somnopentyl, Kyoritsu Seiyaku Co., Tokyo, Japan) at 40 mg/kg body weight and blood samples were collected before the injection and at 1 and 16 hours after the injection for the time course of plasma S1P levels and at 16 hours after injection for the modulation of erythrocyte-related parameters by H_2_DIDS. Then, the S1P levels in the plasma samples and erythrocytes were measured. The mice were euthanized by cervical dislocation without recovery from anesthesia. All the animal experiments were conducted in accordance with the guidelines for Animal Care and were approved by the animal committee of The University of Tokyo.

### Statistical analysis

The results were expressed as the mean ± SEM. Differences between the two groups were evaluated using the Student *t*-test, and differences among more than two groups were assessed using a one-way ANOVA, followed by multiple comparison tests. The sequential differences after the administration of H_2_DIDS were evaluated using paired *t*-test. *P* values less than 0.05 were deemed statistically significant.

## Results

### NaB treatment increased S1P secretion from K562 cells

**Fig 1:**
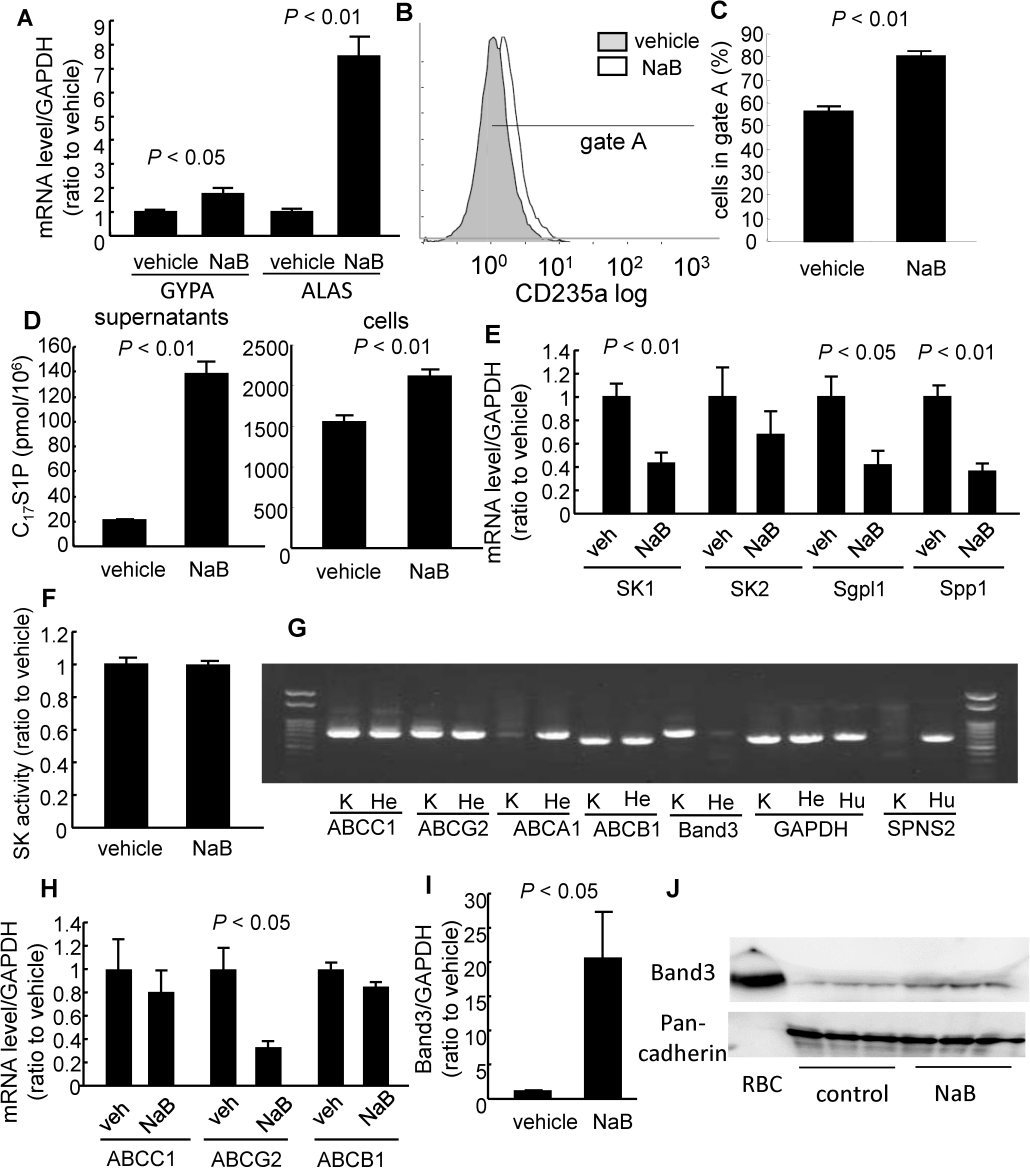
Modulation of S1P homeostasis of K562 cells by sodium butyrate-induced differentiation into erythroblast-like cells. K562 cells were treated with 2 mM sodium butyrate (NaB); 72 hours later, we investigated the modulation of S1P homeostasis. (A) The mRNA levels of GYPA and ALAS were determined using real-time PCR. GAPDH was utilized as an internal control (n = 8/group). (B, C) The expression of CD235a was investigated with a flow cytometer (n = 3/group). (D) C_17_S1P formation assay. NaB-treated or vehicle-treated K562 cells were treated with 10 μM of C_17_sphingosine for 20 minutes. Then, we replaced the supernatant with PBS containing 0.5% BSA and incubated the cells at 37°C for another 20 minutes. Then, the supernatants and cells were collected and used for the C_17_S1P measurements (n = 6/group). (E) The expression of key enzymes in S1P metabolism was determined using real-time PCR. GAPDH was utilized as an internal control (n = 8/group). (F) SK activity assay. The SK activity assay was performed using NaB-treated or vehicle-treated K562 cells (n = 6/group). (G) Reverse transcription PCR was performed using cDNAs prepared from NaB-treated K562 cells (K), HepG2 cells (He), and HUVECs (Hu). (H) The expression of possible S1P transporters was determined using real-time PCR. GAPDH was utilized as an internal control (n = 4/group). (I) Real-time PCR of Band3. GAPDH was utilized as an internal control (n = 8/group). (J) Western blot of Band3 with membranous protein. The whole cell lysate of RBCs (2 μg) was placed as a positive control. Pan-cadherin was utilized as an internal control (n = 3/group).

To search protein(s) involved in the secretion of S1P from erythrocytes, we differentiated K562 cells into erythroblast-like cells by treatment with NaB. Concordant with previous reports using a similar system [24,25], treatment with NaB induced the expression of GYPA and ALAS, which are markers for erythroblasts (Fig 1A). A flow cytometric analysis also revealed the increased expression of CD235a (GYPA) on the NaB-treated K562 cells (Fig 1B and 1C).

Regarding S1P metabolism, we performed a C_17_S1P production assay and found that NaB treatment increased the C_17_S1P levels, especially in the supernatants, together with the cell components (Fig 1D). Despite the increase in C_17_S1P, neither SK1 nor SK2 expression was increased, but rather was decreased, in NaB-treated K562 cells (Fig 1E). We also investigated the activity of SK and found that SK activity was not elevated in NaB-treated K562 cells (Fig 1F), while the expression levels of Sgpl and Spp1, which degrade S1P, were decreased (Fig 1E), which might explain the increased cellular C_17_S1P levels in NaB-treated K562 cells.

At present, several membranous transporters have been proposed to transport S1P out of cells, such as ABCC1, ABCG2, ABCA1, and SPNS2 [26,27,28,29]. Therefore, we next investigated the expression of these transporters in K562 cells. As shown in Fig 1G, reverse transcription PCR performed from cDNA prepared from NaB-treated K562 cells revealed that the expression of ABCA1 and SPNS2 was below our detection limit in NaB-treated K562 cells, while neither ABCC1, ABCB1, nor ABCG2 was increased by treatment with NaB (Fig 1H).

Along with GYPA and ALAS, Band3 is a characteristic protein of erythrocytes and is thought to function as a membranous negative ion exchanger. We found that Band3 was expressed in K562 cells (Fig 1G) and that its expression was increased in NaB-treated K562 cells (Fig 1I and 1J). While the expression of many genes might be modulated by the treatment with NaB, this observation prompted us to examine the possibility that Band3 might affect the efflux of S1P from NaB-treated K562 cells.

### H_2_DIDS treatment inhibited S1P efflux from human erythrocytes

**Fig 2:**
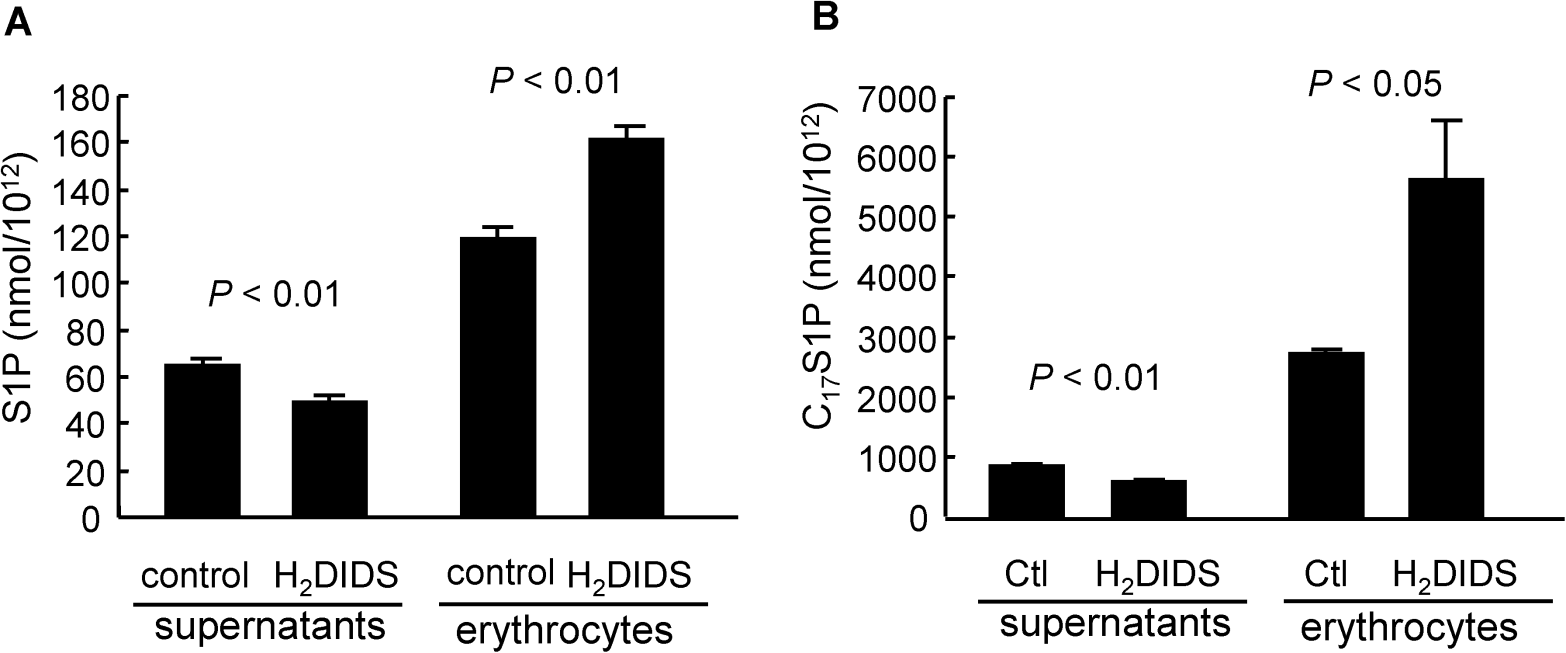
H_2_DIDS treatment inhibited S1P efflux from human erythrocytes to albumin. (A) Erythrocytes were treated with or without 0.1 mM H_2_DIDS and were incubated for 20 minutes with 0.5% BSA. Then, the S1P contents in the supernatants and erythrocytes were determined (n = 4/group). (B) A C_17_S1P formation assay was performed using erythrocytes treated with or without 0.1 mM H_2_DIDS and utilizing 0.5% BSA as an acceptor (n = 4/group).

To investigate whether Band3 affects the efflux of S1P from erythrocytes, we first investigated the involvement of Band3 in the export of S1P from human erythrocytes, using H_2_DIDS to inhibit the function of Band3. As shown in Fig 2A, the treatment with H_2_DIDS successfully decreased the concentration of S1P in the supernatant, while it increased the cellular S1P level in human erythrocytes. Using a C_17_S1P formation assay (in the presence of albumin), we also observed that H_2_DIDS decreased the C_17_S1P efflux, while it increased the cellular C_17_S1P level in human erythrocytes (Fig 2B).

**Fig 3:**
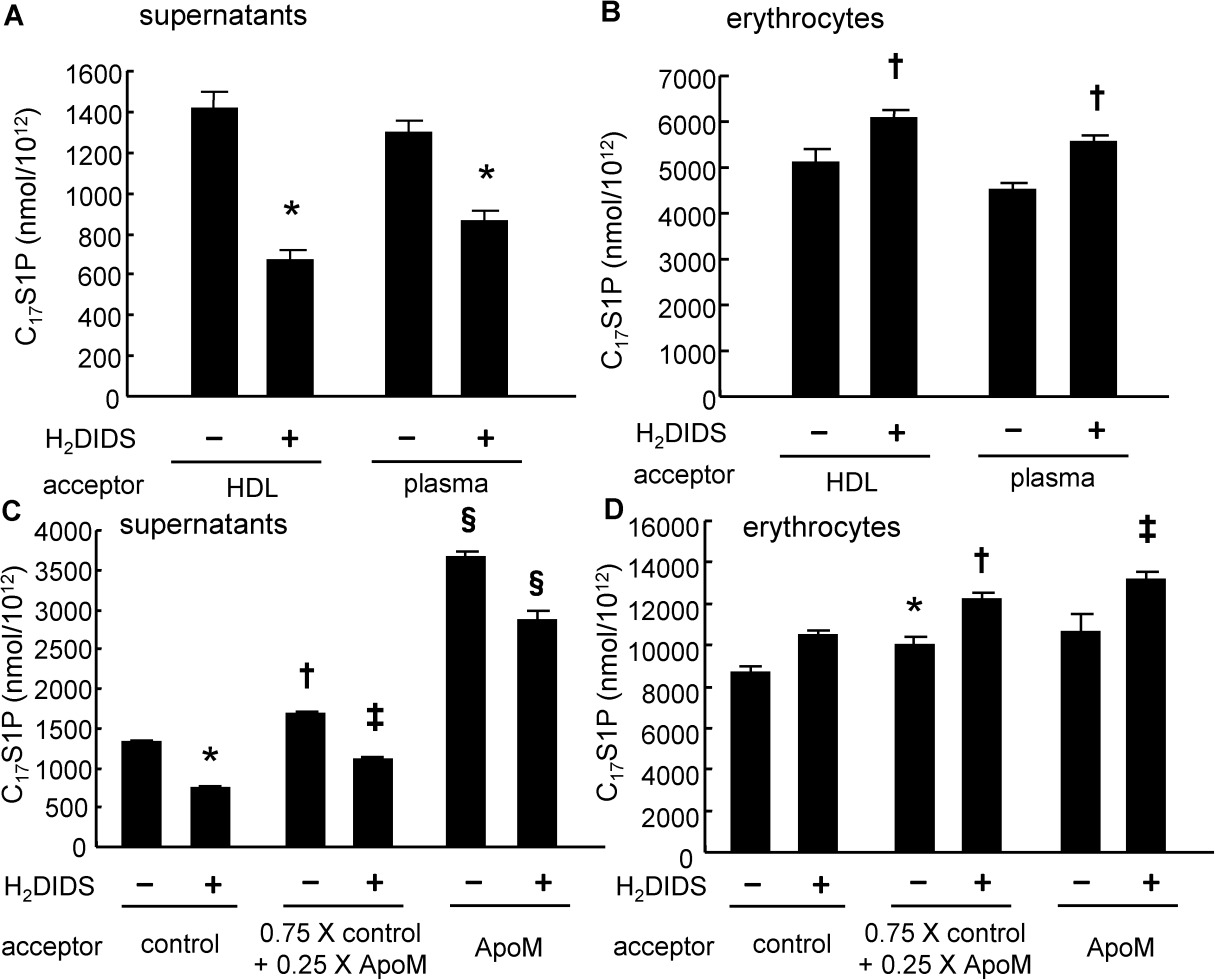
H_2_DIDS treatment inhibited S1P efflux from human erythrocytes to various acceptors. Human washed erythrocytes were treated with or without 0.1 mM H_2_DIDS. Then, a C_17_S1P formation assay was performed utilizing HDL, plasma (A, B), apoM-rich lipoprotein, or control lipoprotein (C, D) as an acceptor. (A, B) A C_17_S1P formation assay was performed using erythrocytes treated with or without 0.1 mM H_2_DIDS and utilizing HDL or plasma as an acceptor (n = 4/group). *P < 0.01 vs. HDL alone and plasma alone, †*P* < 0.05 vs. HDL alone and plasma alone. (C, D) A C_17_S1P formation assay was performed using erythrocytes treated with or without 0.1 mM H_2_DIDS and utilizing various concentrations of apoM-rich lipoprotein and control lipoprotein (n = 4/group). (C) C_17_S1P levels in the supernatants. *P < 0.05 vs. 0.75 X control + 0.25 X ApoM with H_2_DIDS and *P* < 0.01 vs. the other groups, †*P* < 0.05 vs. control alone and *P* < 0.01 vs. other groups, ‡ *P* < 0.01 vs. other groups except control alone, §*P* < 0.01 vs. other groups. (D) C_17_S1P levels in the erythrocytes. *P < 0.05 vs. control alone and ApoM with H_2_DIDS, †*P* < 0.05 vs. ApoM alone and *P* < 0.01 vs. control alone and control with H_2_DIDS, ‡*P* < 0.05 vs. 0.75 X control + 0.25 X ApoM alone, *P* < 0.01 vs. control alone, control with H_2_DIDS, and ApoM alone. Error bars indicate SEM.

Since S1P is carried on both HDL and albumin in plasma, we further investigated the effects of H_2_DIDS on the C_17_S1P efflux from erythrocytes under conditions where HDL or plasma functions as an acceptor for S1P. We observed a decrement in the C_17_S1P efflux and a increment in the cellular C_17_S1P content in erythrocytes treated with H_2_DIDS in both cases (Fig 3A and 3B), similar to the situation when albumin is used as an acceptor. Furthermore, since a recent report suggested that apoM enhances the secretion of S1P from erythrocytes [30], we examined the effects of H_2_DIDS on the C_17_S1P efflux utilizing various concentrations of apoM as an acceptor and observed that apoM significantly enhanced the secretion of C_17_S1P from erythrocytes, which was again inhibited by H_2_DIDS (Fig 3C and 3D).

### Injection of H_2_DIDS reduced plasma S1P levels in mice

**Fig 4:**
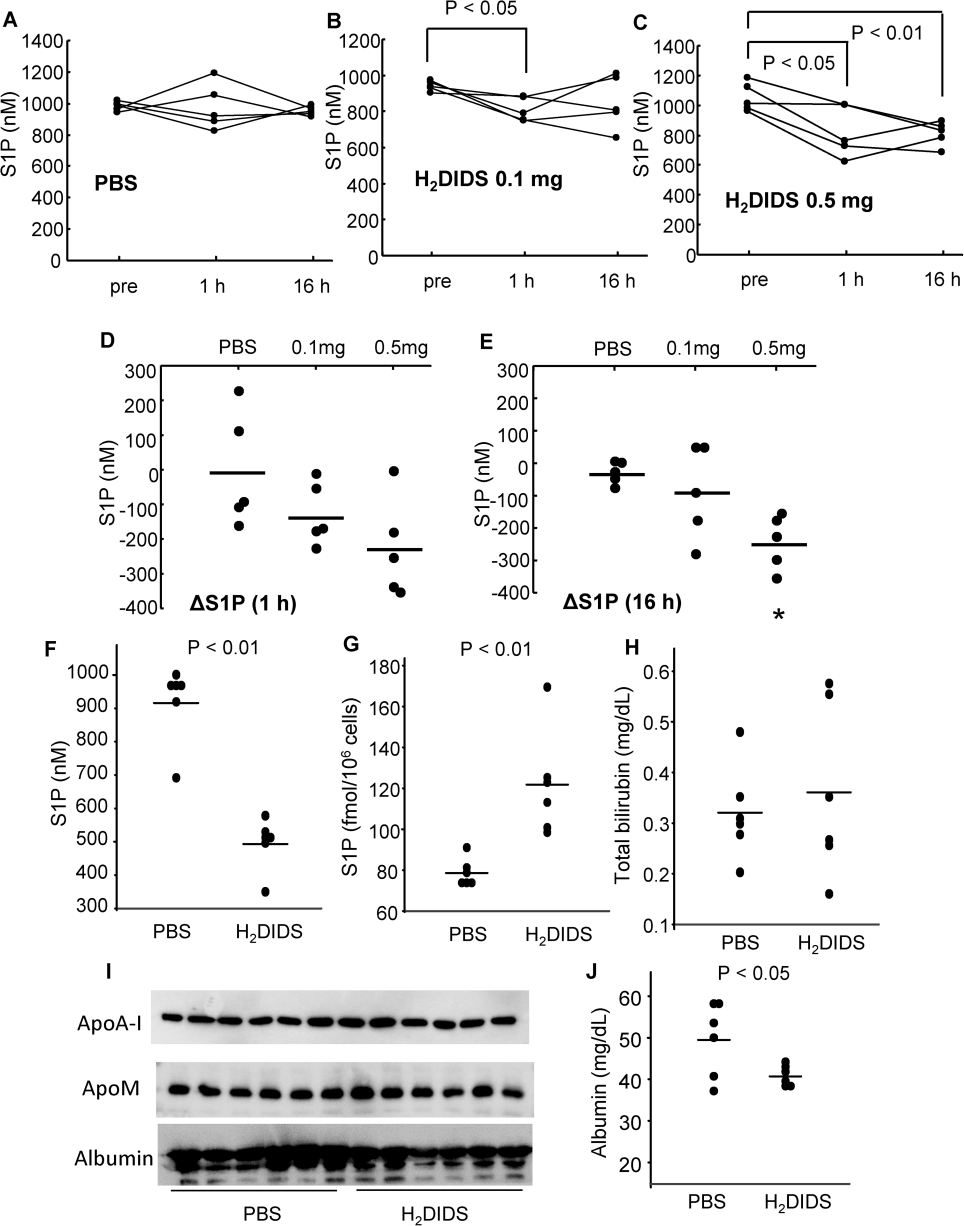
Injection of H_2_DIDS reduced the plasma S1P levels in mice. (A–E) Ten-week-old male mice were divided into three arms; mice were injected with PBS, 0.1 mg of H_2_DIDS, or 0.5 mg of H_2_DIDS. Blood samples were collected before and at 1 and 16 hours after injection. The S1P levels in the plasma samples were then measured (n = 5/group). (A-C) Time course of the plasma S1P levels before and 1 and 16 hours after the administration of H_2_DIDS. (D, E) Changes in the plasma S1P levels after treatment with H_2_DIDS. *P < 0.01, compared with PBS. (F–J) Ten-week-old male mice were administered with PBS or 0.5 mg of H_2_DIDS and after 16 hours the plasma samples were collected (n = 6). (F) Plasma S1P levels. (G) S1P contents in erythrocytes. (H) Total bilirubin levels in the plasma. (I) Western blots of apoA-I, apoM, and albumin with the plasma samples. (J) Plasma albumin levels determined with ELISA.

Next, we injected H_2_DIDS into wild-type mice and tracked the plasma S1P level. Treatment with H_2_DIDS did not modulate the level of hemoglobin in mice; the concentrations of hemoglobin were 13.26 ± 0.79 g/dL, 13.25 ± 0.62 g/dL, and 12.53 ± 0.32 g/dL in mice injected with PBS, 0.1 mg of H_2_DIDS, and 0.5 mg of H_2_DIDS, respectively. Under these conditions, however, we found that treatment with 0.1 mg of H_2_DIDS decreased the plasma S1P level at 1 hour and with 0.5 mg of H_2_DIDS until at 16 hours after administration, compared to the plasma S1P level before the injection (Fig 4A–4C). When we compared the changes in the plasma S1P levels after treatment with H_2_DIDS or PBS, a significant decrease in the plasma S1P levels was observed with the mice treated with 0.5 mg of H_2_DIDS at 16 h after the treatment (Fig 4D and 4E). These results suggest that Band3 is responsible for the exertion of S1P from erythrocytes, at least to some degree.

Furthermore, we investigated whether the treatment with 0.5 mg of H_2_DIDS would affect the hemolysis of erythrocytes or the carrier of S1P (apoM and albumin). As shown in Fig 4F and 4G, we observed that the treatment with H_2_DIDS decreased plasma S1P levels, while increased the S1P contents in erythrocytes. We also found that the total bilirubin levels, a marker for hemolysis, were not affected (Fig 4H) and either the erythrocyte count, MCV, MCH, or MCHC were not different between the mice administered with PBS and H_2_DIDS (857.2 ± 13.8 X 10^4^/μL in PBS vs 797.2 ± 36.1 X 10^4^/μL in H_2_DIDS, 48.60 ± 0.19 fL in PBS vs. 48.67 ± 0.30 fL in H_2_DIDS, 16.52 ± 0.20 pg in PBS vs. 16.95 ± 0.64 pg in H_2_DIDS, and 34.02 ± 0.54 g/dL in PBS vs. 34.85 ± 1.37 g/dL in H_2_DIDS, respectively). Regarding the carrier of S1P, although the apoM levels were not apparently different (Fig 4I), the albumin levels were slightly but significantly lower in the mice treated with H_2_DIDS (Fig 4J).

### Band3 contributed to S1P efflux from K562 cells

**Fig 5:**
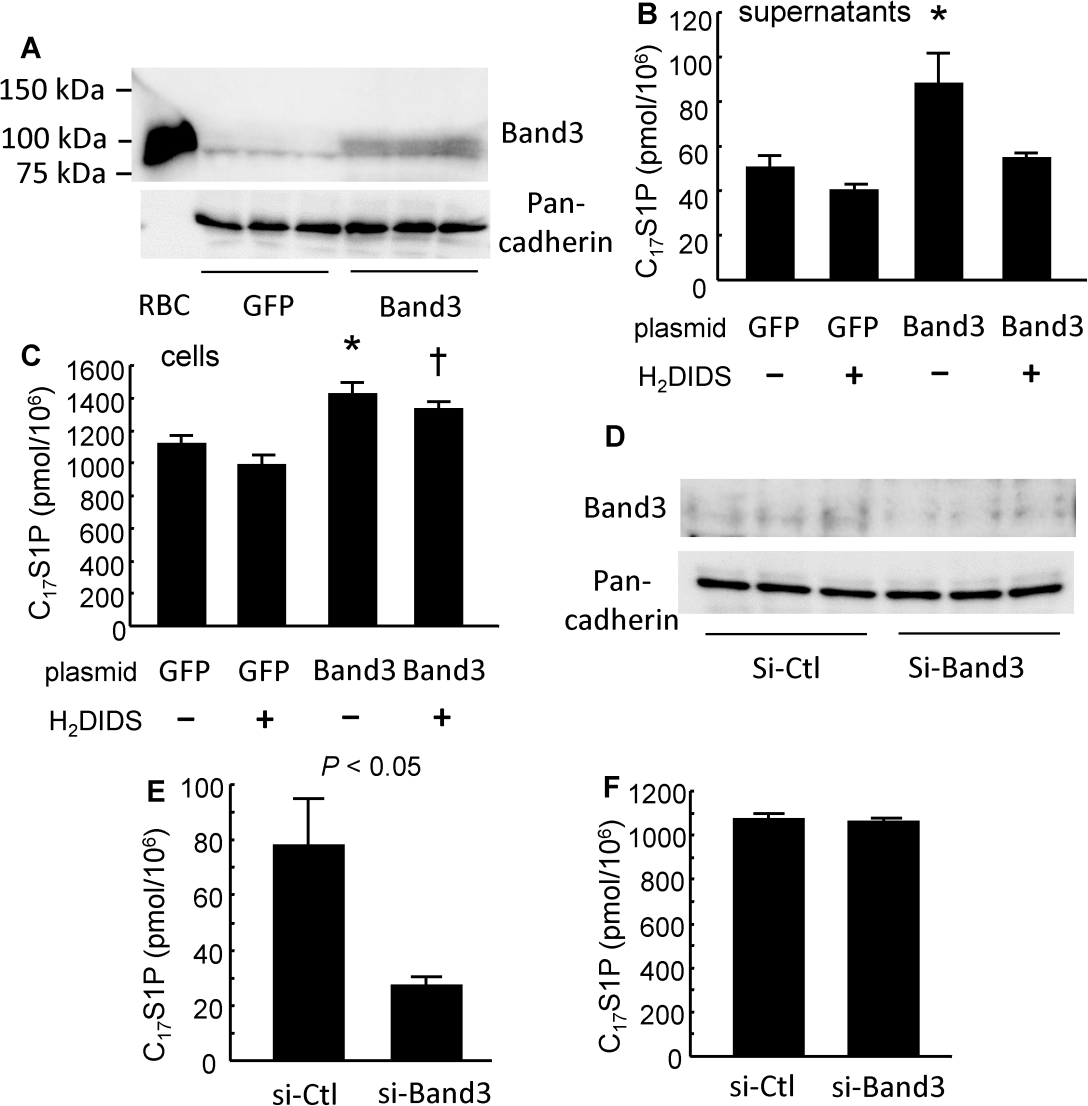
Band3 contributed to S1P efflux from K562 cells. The effects of the overexpression or the knockdown of Band3 on S1P excretion from K562 cells were investigated. (A–C) Band3 plasmid or GFP plasmid was transfected into K562 cells and 48 hours later, a C_17_S1P formation assay was performed with or without 0.1 mM H_2_DIDS. (A) Western blots of Band3 with membranous protein. The whole cell lysate of RBCs (2 μg) was placed as a positive control. Pan-cadherin was utilized as an internal control (n = 3/group). (B, C) C_17_S1P levels in the supernatants (B) and in the K562 cells (C) (n = 5/group). *P < 0.05 vs. GFP alone and GFP with H_2_DIDS. †*P* < 0.05 vs. GFP with H_2_DIDS. (D–F) The knockdown of Band3 was performed using siRNA. Forty-eight hours later, a C_17_S1P formation assay was performed. (D) Western blot of Band3 with membranous protein. Pan-cadherin was utilized as an internal control (n = 3/group). (E, F) the C_17_S1P levels in the medium (E) and in the K562 cells (F) (n = 5/group). Error bars indicate SEM.

Next, we investigated the modulation of C_17_S1P efflux from K562 cells, human chronic myelogenous leukemia cell line, by Band3 using Band3-overexpressing K562 cells and Band3-knockdown K562 cells (Fig 5A and 5D).

As shown in Fig 5B and 5C, the C_17_S1P levels in both supernatants and K562 cells were increased by the overexpression of Band3. H_2_DIDS reversed the C_17_S1P levels only in the supernatants, and not in the K562 cells, increased by the overexpression of Band3. Although Band3 increased the cellular C_17_S1P content, Band3 did not modulate the expression of the enzymes involved in S1P homeostasis (S2A Fig) or the SK activity in K562 cells (S3 Fig).

When we suppressed the expression of Band3 with siRNA, the C_17_S1P efflux from the K562 cells decreased (Fig 5E), while the cellular C_17_S1P content was not modulated (Fig 5F). As was the case with the overexpression of Band3, the knockdown of Band3 did not modulate the expression of proteins related to S1P metabolism (S2B Fig). These results also suggest the possibility that Band3 may have an important role in the excretion of S1P from erythrocytes.

### Band3 did not modulate the efflux of S1P from HEK293 cells

**Fig 6:**
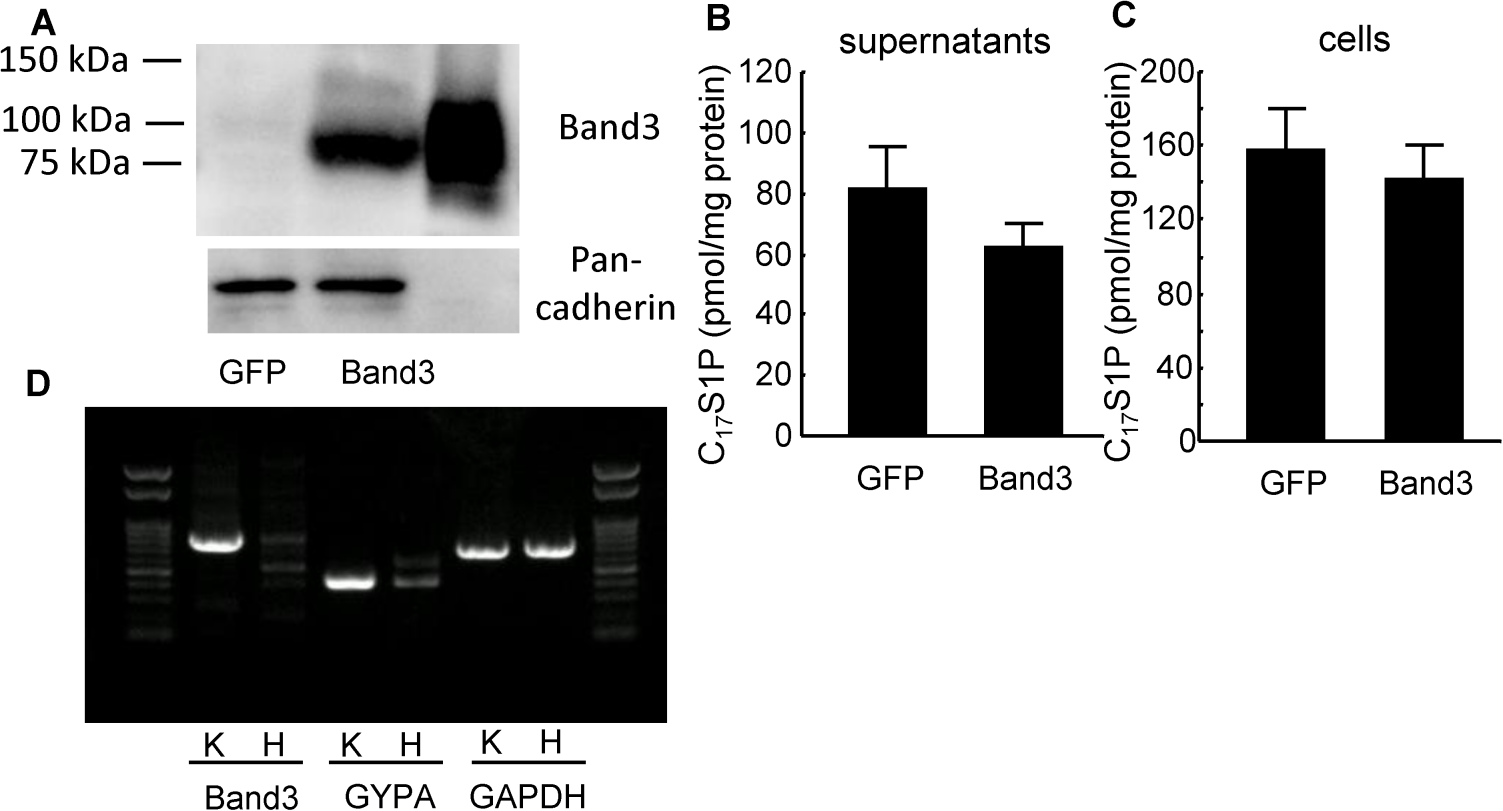
The overexpression of Band3 did not modulate the efflux of S1P from HEK293 cells. The effects of the overexpression of Band3 on S1P excretion from HEK293 cells were investigated. (A) Western blots of Band3 with membranous protein. The whole cell lysate of RBCs (2 μg) was placed as a positive control. Pan-cadherin was utilized as an internal control. (B, C) C_17_S1P levels in the supernatants (B) and in the cellular contents (C) in HEK293 cells (n = 6/group). (D) Reverse transcription PCR was performed using cDNAs prepared from K562 cells (K) and HEK293 cells (H).

Finally, we investigated whether the modulation of S1P efflux by Band3 would be also observed in HEK293 cells (Fig 6A), in which Band3 was hardly expressed (Fig 6D). Contrary to the case with K562 cells, the overexpression of Band3 did not modulate the S1P efflux from HEK293 cells (Fig 6B and 6C).

## Discussion

In this study, to investigate the mechanism for S1P efflux from erythrocytes, we differentiated K562 cells into erythroblast-like cells using NaB and examined the modulation of S1P dynamics. Interestingly, the treatment with NaB increased the secretion of S1P, but the levels of possible candidate transporters proposed in previous studies were not elevated (Fig 1), while we observed that Band3, the most characteristic membranous transporter for erythrocytes, was increased in K562 cells treated with NaB. Therefore, in the present study, we investigated whether Band3 is involved in the S1P efflux from erythrocytes, using H_2_DIDS, an established inhibitor of Band3 [31,32]. We assessed the inhibitory effect of H_2_DIDS in human erythrocytes to confirm the involvement of Band3 in S1P secretion from erythrocytes (Figs 2 and 3), and we also determined that the injection of H_2_DIDS into mice decreased the plasma S1P level (Fig 4). Although plasma albumin levels were decreased in the mice administered with H_2_DIDS (Fig 4J), the decrement of plasma S1P levels might be due to the inhibitory effects of H_2_DIDS on S1P efflux from erythrocytes, but not to the decreased albumin levels, considering that the extent of the decrease in plasma albumin levels was slight and the S1P contents in erythrocytes were increased (Fig 4G). These results were consistent with the idea that the transporter function of Band3 might be at least partly involved in the transport of S1P from erythrocytes. Finally, when we overexpressed Band3 in K562 cells, S1P secretion was accelerated, while S1P secretion was prevented when we suppressed Band3 (Fig 5).

Regarding the association between S1P and erythrocyte-related diseases, two elegant reports recently demonstrated that S1P is elevated in subjects with sickle cell anemia and is involved in the pathogenesis of this disease; S1P might contribute to elevated levels of inflammatory microparticles [33] and promote sickle cell anemia [34]. The mutation of Band3 causes hereditary spherocytosis [35,36]. Although whether the mutant Band3 occurring in hereditary spherocytosis modulates S1P dynamics remains to be investigated, S1P might possibly be involved in the pathogenesis of hereditary spherocytosis. One possible mechanism is that the inhibition of S1P export from erythrocytes may increase the intracellular S1P levels, as shown in Fig 2. Since S1P is an amphiphilic molecule and can possess surfactant characteristics, an elevation in the intracellular or membranous S1P level might promote the hemolysis of erythrocytes. In this study, however, we did not observe any differences in the plasma bilirubin levels or the erythrocyte-related parameters by the administration of H_2_DIDS into wild type mice (Fig 4H). Further study with mice models of erythrocyte-related diseases is needed to elucidate the association between S1P and hereditary spherocytosis.

In addition to the possible involvement of Band3 in S1P transport from erythrocytes, it was interesting that the overexpression of Band3 increased the cellular C_17_S1P content without modulating the key enzymes involved in S1P metabolism or the SK activity (Fig 4C, S2A and S3 Figs). Although the mechanism for the modulation of S1P production by Band3 remains to be identified, the intracellular anion levels might somehow influence the production pathway or the degradation pathway of S1P in living cells, since Band3 is an anion changer and the most abundant membranous protein in erythrocytes. Further study is necessary to elucidate the possibly novel significances of Band3.

It should be kept in mind that H_2_DIDS cannot completely inhibit the secretion of S1P from erythrocytes (Figs 1 and 2). H_2_DIDS is an established specific inhibitor of Band 3 [31,32] and the cross-link between Band3 and H_2_DIDS was recently clarified [37,38]. Considering this potency of H_2_DIDS, we consider the possibility that some other proteins might be involved in the transport of S1P from erythrocytes. Since the volume of erythrocytes is very large, however, the findings from the present study are important in the regulation of S1P homeostasis in vivo.

The main limitation of this study is that we cannot conclude whether Band3 directly transports S1P or Band3 modulates physiological properties of erythrocytes and indirectly influences S1P homeostasis in erythrocytes. The results from the overexpression of Band3 in HEK293 cells (Fig 6A–6C) suggest that Band3 might not directly release S1P, but indirectly modulate the excretion of S1P or that other components, which were not expressed in HEK293 cells, were necessary for Band3 to modulate S1P efflux. Actually, not only Band3 but also GYPA, which reportedly affects the function of Band3 [39], was not well detected in HEK293 cells (Fig 6D). Anyway, the present study suggests that not only the erythrocyte count [20,40], but also the function of erythrocytes through Band3 might determine the plasma S1P level.

In summary, Band3 is involved in the transport of S1P from erythrocytes at least partly and its inhibitor, H_2_DIDS, inhibits S1P secretion from erythrocytes, resulting in a reduced plasma S1P level.

## Supporting information

S1 Fig Scheme for S1P synthesis and degradation in cells. S1P, sphingosine 1-phosphate; SK, sphingosine kinase; Sgpl, S1P lyase; Spp, S1P phosphatase.
(TIF)

S2 Fig Modulation of the expression of enzymes involved in S1P dynamism by the overexpression or knockdown of Band3. Band3 plasmid or GFP plasmid (A) or siRNA against Band3 or control siRNA (B) was transfected into K562 cells; 48 hours later, the expressions of the key enzymes involved in S1P dynamism were determined using real-time PCR. GAPDH was utilized as an internal control (n = 6/group).
(TIF)

S3 Fig Modulation of SK activity by the overexpression of Band3. Band3 plasmid or GFP plasmid was transfected into K562 cells; 48 hours later, the SK activity was measured as described in the *Materials and Methods* section.
(TIF)
