# Supplementary figures and images for: Involvement of Band3 in the efflux of sphingosine 1-phosphate from erythrocytes
Source: PLoS One. 2017 May 11;12(5):e0177543. doi: 10.1371/journal.pone.0177543 (PMC5426782; doi:10.1371/journal.pone.0177543)

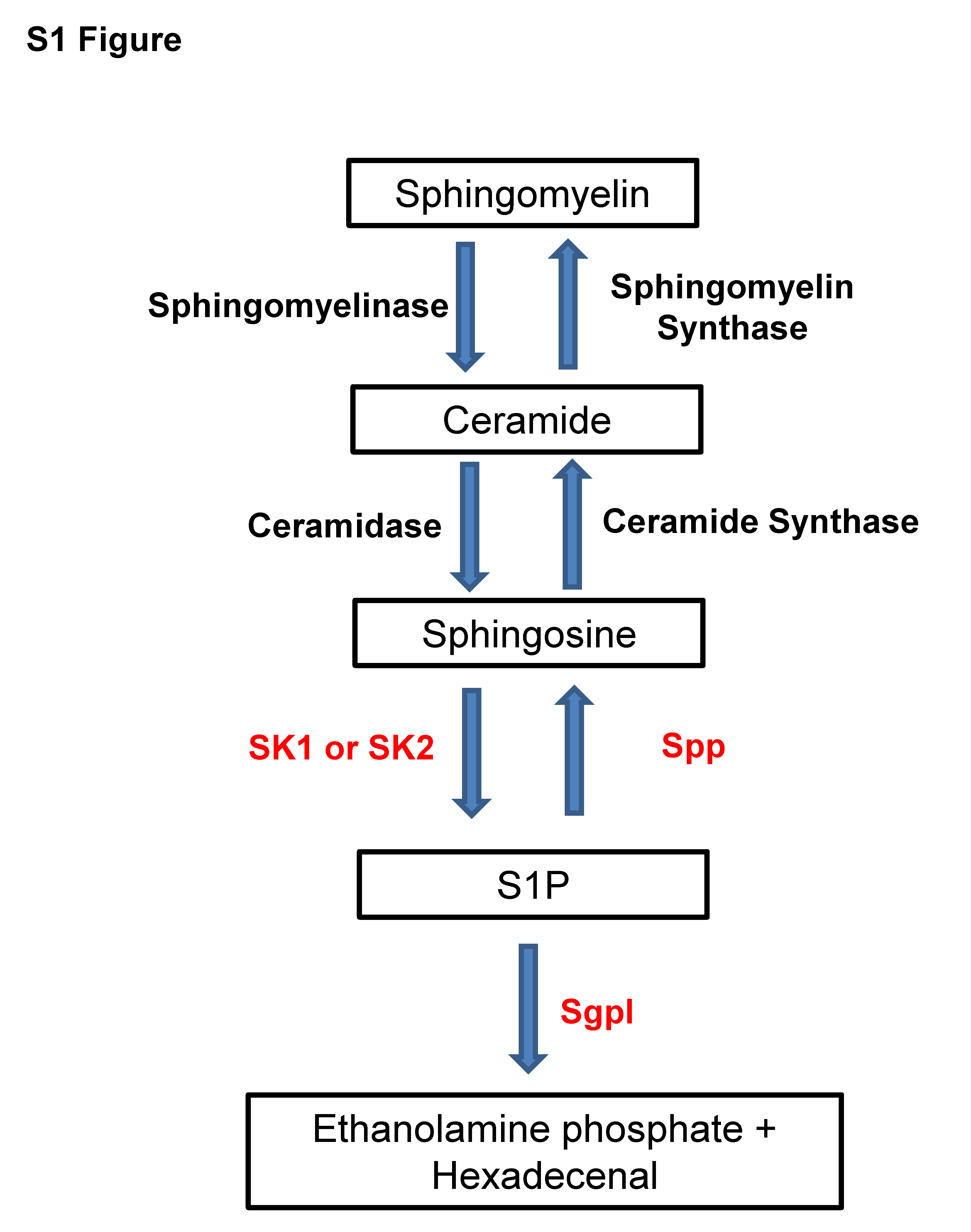

Supplement: S1 Fig — S1P, sphingosine 1-phosphate; SK, sphingosine kinase; Sgpl, S1P lyase; Spp, S1P phosphatase. (TIF) [file pone.0177543.s001.tif]
